# Closing Diagnostic Gaps in Pediatric HIV: Innovations in Point-of-Care and Digital Monitoring with an Asia–Pacific Implementation Lens—A Systematic Review

**DOI:** 10.3390/diagnostics16091306

**Published:** 2026-04-27

**Authors:** Miao-Chiu Hung, Hsihsien Wei

**Affiliations:** 1 Division of Infectious Diseases, Department of Pediatrics, Taipei Veterans General Hospital, Taipei 112201, Taiwan; 2 Pediatrics, School of Medicine, National Yang Ming Chiao Tung University, Taipei 112304, Taiwan; 3 Department of Pediatrics, Taipei Tzu Chi Hospital, Buddhist Tzu Chi Medical Foundation, New Taipei City 231016, Taiwan; 4 Institute of Emergency and Critical Care Medicine, National Yang Ming Chiao Tung University, Taipei 112304, Taiwan

**Keywords:** pediatric HIV, early infant diagnosis, point-of-care testing, viral load, dried blood spot, GeneXpert, digital health, Asia–Pacific, implementation, systematic review, INPLASY

## Abstract

**Background/Objectives:** Pediatric HIV case-finding and monitoring remain constrained by delayed early infant diagnosis (EID), loss to follow-up, and limited viral load (VL) testing—challenges particularly consequential in the operationally diverse Asia–Pacific region. We systematically reviewed innovations in point-of-care (POC) and near-patient HIV diagnostics and digital monitoring relevant to children and adolescents. **Methods:** Following a registered protocol (INPLASY2025110058) and PRISMA 2020 guidance, we searched PubMed, EMBASE, Cochrane Library, and WHO Global Index Medicus for studies on POC/near-patient EID and VL testing, dried blood spot (DBS) workflows, and digital monitoring tools. Risk of bias was assessed using RoB 2, QUADAS-2, and MMAT. **Results:** Fifty-three primary studies were included (39 sub-Saharan Africa, 12 Asia–Pacific, 1 multi-country/global, 1 Americas/Caribbean). Patient selection and flow/timing were common limitations in diagnostic accuracy studies; sample representativeness and nonresponse bias were frequent concerns in implementation studies. The most consistent benefits of POC EID and near-patient VL testing were shorter turnaround times and improved cascade completion when paired with quality assurance and connectivity. **Conclusions:** POC diagnostics and digital monitoring can help close pediatric HIV cascade gaps, though evidence derives predominantly from sub-Saharan Africa. Impact depends on implementation design. Asia–Pacific programs should prioritize generating context-specific evidence alongside the adaptation of established lessons.

## 1. Introduction

Despite major gains in the prevention of vertical transmission and antiretroviral therapy (ART) coverage, children and adolescents remain the population most likely to be diagnosed late and least likely to achieve durable viral suppression. Global reporting continues to show persistent pediatric diagnostic and treatment gaps, with slower progress towards the UNAIDS targets for children than for adults [1,2,3,4]. Across settings, the pediatric cascade is constrained by three interdependent failures: (i) missed opportunities to diagnose maternal HIV during pregnancy and breastfeeding; (ii) delayed virological testing for HIV-exposed infants; and (iii) limited access to timely viral load (VL) monitoring to confirm treatment success and trigger regimen switches when needed [5,6,7]. WHO guidance for pediatric treatment and care emphasizes early identification, rapid ART initiation, and age-appropriate regimens and follow-up, implemented through a simplified public health approach that depends on reliable monitoring systems [8,9]. Where VL monitoring is limited, programs may fall back on clinical and immunologic signals, but this approach is less sensitive for early treatment failure and is increasingly discouraged in favor of routine VL access [7]. Earlier POC immunologic technologies (e.g., CD4) remain relevant as complementary tools in some rural settings but do not substitute for virological monitoring [10]. Global HIV statistics from UNAIDS also continue to document that pediatric coverage and outcomes lag behind adult benchmarks, reinforcing the need for diagnostics that accelerate the entire cascade [1].

The Asia–Pacific region illustrates both the urgency and the complexity of the pediatric diagnostic challenge. The region encompasses countries with mature laboratory networks and high antenatal care (ANC) coverage—such as Thailand and parts of China—alongside geographically dispersed island and mountainous settings in the Pacific, Papua New Guinea, and highland areas of Southeast Asia, where specimen transport, cold-chain maintenance, and linkage to care are intrinsically difficult.

Several features of the Asia–Pacific context create implementation challenges that are distinct from those documented in the sub-Saharan African literature that dominates the evidence base. Firstly, epidemic profiles in the region are heterogeneous: some countries have concentrated epidemics among key populations (e.g., people who inject drugs, sex workers, men who have sex with men), whereas others have low generalized prevalence with geographically focal transmission—patterns that affect the yield and cost-effectiveness of different testing strategies [11,12]. Secondly, health system organization varies from centralized national laboratory networks capable of high-throughput molecular testing to primary care systems with limited infrastructure, creating a need for hybrid diagnostic models that combine POC testing at high-volume facilities with optimized specimen referral for low-volume sites [13,14]. Thirdly, cultural and structural barriers—including HIV-related stigma, gender-based barriers to maternal health-seeking, and fragmented service delivery across maternal–child health and HIV programs—differ in form and intensity from those in African settings and may require region-specific implementation strategies [15]. These considerations justify a dedicated synthesis that applies an Asia–Pacific implementation lens to the available evidence, rather than assuming direct transferability of solutions designed for high-burden African contexts.

From a diagnostic systems perspective, pediatric HIV programs face a distinctive set of constraints that differ from adult testing. In infants, serological assays are unreliable because of transplacentally acquired maternal antibodies; virological tests are therefore required for definitive diagnosis [16,17]. In adolescents, the clinical challenge often shifts from diagnosis to long-term retention and adherence, where service delivery models must be youth-friendly and confidentiality-protecting [18,19]. Across age groups, the clinical value of an accurate test is realized only if results are delivered, documented, and acted upon—an insight reflected in WHO strategic information guidance and in program reports describing missed opportunities for pediatric VL action [7,20].

To address these gaps, global guidance increasingly prioritizes diagnostics that shorten the time between specimen collection, result availability, and clinical action. WHO guidelines recommend virological testing of all HIV-exposed infants at birth (where feasible) or at 4–6 weeks, with repeat testing aligned to ongoing exposure and breastfeeding status, and routine VL monitoring after ART initiation [16,17]. At the same time, the World Health Organization has articulated target product profiles (TPPs) for point-of-care (POC) EID technologies that could enable the decentralization of virological testing to maternity wards and primary care clinics [21]. The HIV diagnostics “technology landscape” also indicates rapid diversification of near-patient platforms, including multi-disease instruments that can be leveraged through integrated laboratory and supply chain systems [13,14].

Diagnostic innovations alone, however, rarely translate into improved outcomes unless paired with implementation strategies that address the full diagnostic value chain: test placement, training and workflow design, specimen transport (when POC is not available), connectivity, quality assurance, and mechanisms that ensure that results trigger appropriate action. Programmatic experience and implementation reviews from resource-limited settings emphasize that “POC” does not automatically mean “patient-impacting” without deliberate design for linkage and retention [22,23,24]. Digital tools—ranging from electronic laboratory information systems and connectivity middleware to mHealth adherence support—may strengthen this chain by improving the visibility of test orders, results, follow-up steps, and adherence risks [7,14,25,26,27].

Given the accelerating pace of innovation and the heterogeneity of health system constraints across Asia–Pacific settings, decision-makers require an evidence synthesis that is both technically grounded (diagnostic performance, operational feasibility) and implementation-focused (turnaround time, linkage to care, and sustainability) [19,22,23,24].

Review question: In infants, children, and adolescents (0–19 years) living with HIV or exposed to HIV, what POC/near-patient diagnostic and digital monitoring innovations improve (i) timely HIV diagnosis, (ii) timely ART initiation, and (iii) VL monitoring and treatment action, with specific attention to implementation feasibility in the Asia–Pacific region?

Objectives: (1) To summarize the diagnostic performance and clinical impact of POC/near-patient virological testing for pediatric EID and VL monitoring; (2) to characterize the geographic distribution and design features of the available evidence base, including Asia–Pacific representation; (3) to synthesize implementation enablers and risks (quality assurance, connectivity, and program design) relevant to regional scale-up; and (4) to provide a practical, evidence-informed checklist for implementers.

## 2. Materials and Methods

### 2.1. Protocol and Registration

This review was conducted according to a prespecified protocol registered in INPLASY (INPLASY2025110058; https://inplasy.com/inplasy-2025-11-0058/, accessed on 23 April 2026). Compared with the registered protocol, the search window was extended from 2015 to 2012 to accommodate older foundational pediatric EID implementation studies. This modification was made after protocol registration but before database searches were executed and before any screening of results; it was based on recognition during search strategy finalization that key foundational EID implementation studies (e.g., early POC EID field evaluations and DBS workflow analyses) predated the originally planned 2015 start date, and their exclusion would weaken the evidence synthesis. Additionally, the planned appraisal framework for non-randomized implementation studies was updated from RoBANS to MMAT (2018) to better reflect the heterogeneity of the included evidence. We acknowledge that these protocol deviations were not prospectively updated in the INPLASY registry, which we note as a limitation. Finally, two primary studies from the LIFE study (Jani et al., 2025 [28]; Lwilla et al., 2025 [29]) were identified during peer review and added to the synthesis post hoc; these studies were published within the original search window but were inadvertently missed during the initial screening process. We report methods and results in accordance with PRISMA 2020 guidance (see in Supplementary Materials) [30].

### 2.2. Eligibility Criteria

We included peer-reviewed original studies and programmatic reports that evaluated POC or near-patient HIV diagnostic technologies (EID and/or VL), dried blood spot workflows, or digital monitoring tools in pediatric and adolescent populations (0–19 years), published from 1 January 2012 to 1 November 2025. Eligible study designs included randomized controlled trials, quasi-experimental evaluations, diagnostic accuracy studies, observational cohort or cross-sectional studies, mixed-methods implementation evaluations, and structured program reports. We excluded commentaries, editorials, narrative reviews without original data, conference abstracts without accompanying full-text publications, studies enrolling exclusively adult populations (>19 years) without disaggregated pediatric data, and reports focused exclusively on legacy assay versions or diagnostic approaches not aligned with contemporary WHO normative guidance. Specifically, we operationalized this criterion by reference to contemporary WHO normative documents: this judgment was informed by contemporary WHO recommendations for pediatric HIV diagnosis and monitoring [31] and, where relevant, by WHO prequalification documentation [32]. We did not use WHO listing status alone as proof of real-world clinical use or non-use; studies evaluating currently relevant platforms or their direct predecessors were retained regardless of brand or product code changes over time. Technologies that remain in limited programmatic use as complementary tools (e.g., POC CD4 testing) were retained where they contributed to the understanding of cascade completion or served as comparators.

### 2.3. Information Sources and Search Strategy

We searched PubMed/MEDLINE, EMBASE, the Cochrane Library, and WHO Global Index Medicus for studies published from 1 January 2012 to 1 November 2025. We complemented database searches with targeted searches of major agency reports and guidance documents, including UNAIDS, WHO, UNICEF, and PEPFAR pediatric reporting. Search concepts combined pediatric HIV, early infant diagnosis, viral load monitoring, point-of-care and near-patient testing, dried blood spot workflows, and digital/connected diagnostics (examples of search terms: “early infant diagnosis”, “point-of-care”, “GeneXpert”, “Xpert HIV-1 Qual”, “viral load”, “SAMBA”, “dried blood spot”, “connectivity”, “SMS”, “mHealth”).

### 2.4. Study Selection

**Figure 1:**
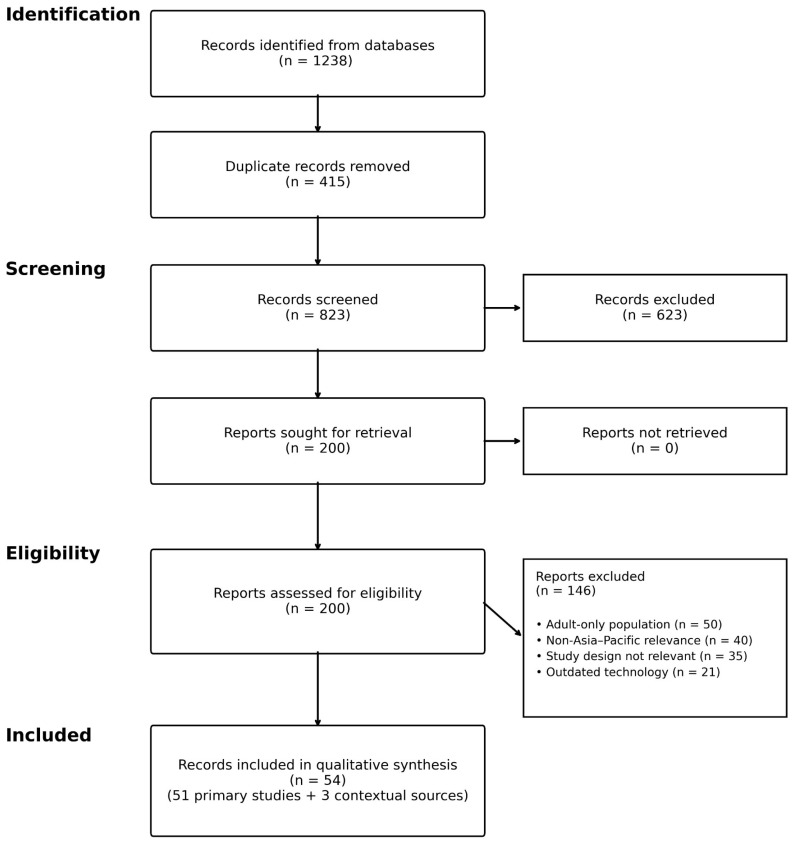
PRISMA 2020 flow diagram for study selection. Reports focused exclusively on obsolete technologies, as defined in Section 2.2, were excluded during full-text review within the full-text exclusion category shown in the figure. The final qualitative synthesis comprised 56 records, including 53 primary studies and 3 contextual sources retained to support the narrative synthesis.

After de-duplication, titles and abstracts were screened for relevance. Full texts were retrieved for potentially eligible studies and assessed against eligibility criteria. Uncertain classifications were resolved through re-review and consultation with the wider research team. The PRISMA flow diagram (Figure 1) summarizes the selection process and final inclusion counts.

### 2.5. Data Extraction

For each included study, we extracted: publication year; country/setting; study design; population; technology/workflow (EID, VL, DBS, digital/system intervention); comparator (if any); and outcomes (diagnostic performance; turnaround time; completion of testing and communication steps; ART initiation within prespecified time windows; VL monitoring and time-to-action metrics). Study characteristics are presented in Supplementary Table S3.

### 2.6. Risk-of-Bias Assessment

Randomized and quasi-experimental evaluations were assessed with the RoB 2 [33] framework. Diagnostic accuracy studies evaluating an index test against a reference standard were appraised using QUADAS-2 [34] domains (patient selection, index test, reference standard, and flow/timing). Implementation and service delivery studies that did not report formal test accuracy endpoints were appraised using the Mixed Methods Appraisal Tool (MMAT) [35]. Domain-level summaries are provided in Table 4 (RoB 2), Table 5 (QUADAS-2), and Table 6 (MMAT). Study-level domain judgments are provided as Supplementary Tables S1 and S2a,b.

### 2.7. Synthesis Approach

We conducted a structured narrative synthesis and used descriptive statistics for study characteristics. Throughout the results, we distinguish findings derived directly from the 53 primary studies included in this review from quantitative estimates borrowed from external sources; the latter are identified with explicit attribution (e.g., “a previously published meta-analysis [36]”) to prevent conflation. Where robust pooled quantitative evidence was available from high-quality systematic reviews, we summarized the relevant pooled estimates (e.g., ART initiation outcomes following POC EID) [36].

## 3. Results

### 3.1. Study Selection

The database and report searches identified 1238 records. After removing 415 duplicates, 823 records were screened and 200 full-text reports were assessed for eligibility. A total of 51 primary studies met the inclusion criteria through the systematic search; two additional primary studies (Jani et al., 2025 [28]; Lwilla et al., 2025 [29]) from the LIFE study were identified during revision following Academic Editor feedback and added to the synthesis, bringing the total to 53 primary studies (Figure 1). Three further records (one meta-analysis [36] and two guidance frameworks [16,37]) were retained as contextual references but excluded from the risk-of-bias appraisal set. No reports were unretrievable after full-text screening.

### 3.2. Characteristics of Included Studies and Geographic Distribution

Across the 53 included primary studies, the evidence base was geographically concentrated in sub-Saharan Africa (39/53), with limited but meaningful representation from Asia–Pacific settings (12/53) and a small number of multi-country/global analyses (1/53). Asia–Pacific evidence originated from China (*n* = 2), India (*n* = 3), Thailand (*n* = 2), Papua New Guinea (*n* = 1), Myanmar and Papua New Guinea (*n* = 1), Vietnam (*n* = 1), Cambodia (*n* = 1), and Kazakhstan (*n* = 1). Most studies focused on HIV-exposed infants and EID workflows (35/53), reflecting the clinical priority of diagnosing infection during infancy and the historically low coverage of timely virological testing in many programs [4,37]. Fewer studies focused primarily on older children (4/53) or adolescents (2/53), despite increasing recognition that adolescent retention and adherence are major determinants of long-term viral suppression [18,19]. Eleven studies addressed mixed pediatric populations or health system interventions spanning multiple age bands. In terms of innovation focus, 29 studies evaluated POC EID technologies and/or EID workflow redesign; eight evaluated POC/near-patient VL monitoring; 10 evaluated digital and health system interventions supporting testing and/or monitoring cascades; and six assessed complementary approaches (e.g., POC immunologic monitoring such as CD4, demand-side incentives, or linkage strategies) [10,38]. Table 1 presents the cross-tabulation of innovations by region, highlighting that Asia–Pacific studies were dominated by EID evaluations, with sparse regional evidence on near-patient VL and digital cascade tools. Full study-level characteristics and extracted outcomes are provided in Supplementary Table S3.

### 3.3. Innovations for Pediatric and Infant HIV Diagnosis

#### 3.3.1. Point-of-Care Nucleic Acid Testing for EID

POC EID platforms aim to shift virological testing from centralized laboratories to service delivery points where HIV-exposed infants are seen, including maternity wards, immunization clinics, and outpatient child health services. The most widely studied POC EID approaches used cartridge-based nucleic acid amplification tests deployed on multi-disease platforms. Across evaluations, common operational endpoints included same-day result availability, reduced turnaround time relative to conventional PCR pathways, and improved completion of cascade steps (sample collection → testing → result delivery → clinical action) [22,39,40].

More broadly, in a previously published systematic review and meta-analysis (not conducted as part of the present review), Luo et al. [36] reported that POC EID substantially improved timely ART initiation, with a notably higher proportion of infants initiating ART within 60 days of diagnosis compared with conventional laboratory workflows. In that externally derived pooled analysis, the proportion of infants initiating ART within 60 days of diagnosis was approximately 90% with POC EID compared with approximately 52% under conventional laboratory pathways, and the median time to ART initiation was markedly shorter (on the order of weeks) in POC models [36]. These pooled estimates should be interpreted in the context of the source meta-analysis’s included studies, which were predominantly from sub-Saharan African settings. Consistent with these pooled findings, programmatic implementation of POC EID in Mozambique demonstrated that same-day diagnosis at health facilities significantly increased the proportion of HIV-positive infants initiating ART, providing direct single-country evidence that POC testing can accelerate the diagnosis-to-treatment transition when embedded in routine care [41].

Diagnostic performance is a prerequisite for safe decentralization. Laboratory and field evaluations of POC EID assays (including Alere q and cartridge-based platforms) generally reported high agreement with reference laboratory PCR, while also highlighting program-critical needs: standardized training, external quality assurance, and protocols for confirmatory testing and management of discordant results [22,42,43]. In settings with low pretest probability, even small changes in assay specificity can drive programmatic complexity through false-positive follow-up investigations, reinforcing the importance of quality systems [22].

Detailed innovation-level characteristics and programmatic implications are summarized in Table 2; cascade-level gaps and suggested monitoring indicators are presented in Table 3.

#### 3.3.2. Birth Testing and Repeat Testing Strategies

Evidence from birth POC testing in South Africa (noting that this evidence is from a high-burden African setting and may not directly reflect operational conditions in Asia–Pacific countries with lower prevalence or different health system structures) demonstrated that birth testing is feasible and can detect infections that would otherwise be missed or detected late, but outcomes depend on implementation design [39].

The most substantial evidence on birth POC testing comes from the LIFE (Long-term Impact on inFant hEalth) study, a cluster-randomized trial conducted at 28 health facilities in Mozambique and Tanzania. In one trial arm, Jani et al. [28] evaluated POC test-and-treat at birth (providing same-day EID and immediate ART initiation for HIV-positive infants) compared with standard-of-care testing at 4–8 weeks. The intervention substantially shortened time to ART initiation and was associated with a clinically relevant reduction in early mortality. Viral suppression was higher in the intervention group at 4–8 weeks and at 18 months; however, the overall 18-month composite clinical outcome was not significantly different between study arms, and viral suppression remained poor overall [28]. In a companion analysis from the same trial, Lwilla et al. [29] demonstrated that POC maternal viral load testing at delivery substantially improved the identification of infants at high risk of vertical transmission compared with clinical criteria alone and, in Tanzania—where enhanced postnatal prophylaxis was risk-stratified rather than universal—improved the proportion of high-risk infants receiving appropriate prophylaxis, although prophylaxis remained incomplete [29]. Together, these LIFE study findings provide the strongest trial-level evidence to date that POC testing at birth can improve early risk stratification and proximal cascade outcomes, while underscoring that technology alone is insufficient to guarantee sustained 18-month clinical benefit without effective treatment and retention systems.

#### 3.3.3. Optimizing Dried Blood Spot (DBS) Workflows and Diagnostic Networks

In many Asia–Pacific settings, DBS remains the dominant specimen pathway for virological testing because it tolerates ambient transport and can leverage centralized laboratory capacity. However, DBS workflows are often undermined by specimen transport delays, batching practices, and fragmented result return systems—factors that can negate the clinical value of a technically accurate test [37,44]. Programmatic syntheses emphasize that “turnaround time” is not solely a laboratory metric; it is a system property that reflects the entire value chain from sample to action [22,23]. Connectivity solutions—electronic test ordering, automated result transmission, and dashboards tracking overdue results—can shorten delays and reduce losses between steps [7,14]. Technology landscape analyses further suggest that multi-disease diagnostic platforms could be leveraged to improve resilience, provided that procurement, maintenance, and quality assurance systems are strengthened [13,24].

Table 2 summarizes the major diagnostic innovations and their programmatic advantages and limitations; Table 3 maps these innovations to common cascade gaps that must be addressed for patient-level benefit.

### 3.4. Innovations for Viral Load Monitoring and Treatment Action

VL monitoring is central to confirming ART effectiveness, identifying adherence problems, and triggering regimen switches. Yet VL coverage is consistently lower among children and adolescents than among adults, and public reporting continues to highlight weaker suppression in pediatric populations than in adult cohorts [6,7,50]. In centralized testing models, the time from sample collection to clinical action can also be prolonged [7]. POC and near-patient VL platforms aim to shorten the interval between viremia detection and treatment action.

A randomized evaluation comparing clinic-based SAMBA-II testing with centralized VL testing among children reported improved operational performance (shorter turnaround and more timely clinical decision-making) without compromising analytical validity [45]. However, the Opt4Kids trial in Kenya—which evaluated POC VL combined with targeted drug resistance testing among children on ART—found improved operational turnaround but no significant difference in viral suppression, underscoring that POC technology alone, without systems to act on results and address underlying resistance, may be insufficient to improve clinical outcomes [51]. Early decentralization experiences from Rwanda similarly demonstrated feasibility but emphasized that performance depends on human resources, supply continuity, and data connectivity to ensure that VL results trigger action [47]. Multisite evaluations of near-patient VL technologies have generally shown high agreement with laboratory standards and have highlighted the importance of deployment strategies that prioritize high-volume sites and established specimen transport for lower-volume facilities [46,52].

In practice, many programs in the region already operate GeneXpert instruments primarily for tuberculosis. Implementation experience indicates that integrating HIV assays onto existing multi-disease platforms can be feasible, but it requires explicit governance for cross-program scheduling, cartridge forecasting, instrument maintenance, and quality management [14]. These considerations are particularly relevant for Asia–Pacific settings with dispersed service delivery points, where leveraging existing diagnostic networks may be more realistic than creating parallel pediatric VL infrastructure from scratch.

### 3.5. Digital and System Innovations Across the Pediatric Cascade

Digital tools appear most impactful when they address specific failure points: incomplete test orders, delayed result reporting, missed appointments, and weak visibility of the cascade at facility and district levels [7,14]. Electronic adherence-monitoring tools, such as pill containers and related digital supports, can improve the visibility of pediatric treatment behavior and help identify children who may need additional follow-up [25]. More broadly, studies evaluating digital adherence and psychosocial support for adolescents indicate potential benefits for engagement, retention, and selected viral suppression outcomes, particularly when interventions are contextually adapted and supported by human follow-up rather than delivered as purely automated messaging [26,27,27]. WHO guidance and broader implementation reviews emphasize, however, that digital tools are not substitutes for functioning health systems and that acceptability and feasibility remain more commonly demonstrated than sustainability or scalability [7,53,54].

Demand-side and linkage interventions also interact with diagnostic performance. Conditional cash transfers and community-based linkage approaches have been evaluated to improve uptake of pediatric services and retention, illustrating that diagnostic innovations may require complementary behavioral and service delivery strategies to realize health impact [38,49]. WHO and UNICEF guidance highlight that the integration of pediatric HIV testing into child health platforms (immunization, malnutrition services, inpatient wards) and index case testing can substantially increase case-finding yield when implemented ethically and with safeguards [55,56]. In adolescent populations, HIV self-testing policies and caregiver-assisted approaches may complement facility-based services, but they require clear linkage pathways and the protection of confidentiality [18,48].

**Figure 2:**
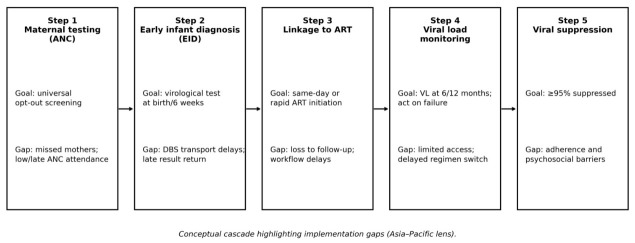
Pediatric HIV testing and monitoring cascade with common programmatic gaps addressed by POC diagnostics and digital monitoring innovations.

These digital and service delivery innovations are directly relevant to the cascade gaps summarized in Figure 2. In particular, they address the transitions most likely to fail in routine care: diagnosis-to-ART initiation and VL result-to-clinical action. Differentiated service delivery frameworks for children and adolescents provide an additional lens for tailoring visit schedules and monitoring intensity according to clinical stability and social context [19].

### 3.6. Cascade Gap Synthesis and Rationale for Figure 2

Figure 2 presents a five-step schematic of the pediatric HIV testing and monitoring cascade and the most common failure points targeted by the innovations in this review. The cascade structure reflects WHO testing service and treatment monitoring recommendations, which define maternal testing as the entry point for prevention and infant follow-up, and virological testing/VL monitoring as the core diagnostic technologies for infants and children on ART [7,16,17]. Firstly, gaps in maternal testing and retesting (during late pregnancy and breastfeeding) continue to drive missed identification of HIV-exposed infants, a priority emphasized in EMTCT and triple-elimination roadmaps for the Asia–Pacific region [11,12]. Secondly, EID gaps arise from delayed specimen movement and slow result return—system weaknesses repeatedly highlighted by global pediatric reports and EID strategic frameworks [4,37]. Thirdly, even when diagnosis occurs, the diagnosis-to-treatment transition is vulnerable: pooled evidence indicates that POC EID improves timely ART initiation, but only when linkage systems are designed to act on results rapidly [23,36]. Fourthly, ongoing monitoring gaps reflect limited access to VL testing and delays in treatment action following detectable VL, which WHO strategic information guidance identifies as a key driver of suboptimal suppression [7]. Finally, long-term viral suppression in children and adolescents is shaped not only by monitoring access but also by adherence and service delivery models, including differentiated services and adolescent support interventions [19,26,50,57]. By explicitly mapping innovations to these cascade steps, Figure 2 is intended to make clear where evidence supports impact and where residual implementation gaps remain.

### 3.7. Risk of Bias

The risk-of-bias assessment results are summarized in Table 4 (RoB 2 for randomized and quasi-experimental evaluations; *n* = 10), Table 5 (QUADAS-2 for diagnostic accuracy studies; *n* = 11), and Table 6 (MMAT for implementation and service delivery studies; *n* = 32). Among the randomized and quasi-experimental evaluations, “some concerns” were most frequent for deviations from intended interventions, while outcome measurement and reporting domains were predominantly low risk. Among diagnostic accuracy studies, the most common limitations were unclear or high risk related to patient selection and flow/timing, emphasizing the importance of prospective recruitment and standardized reference testing protocols [22]. Among the implementation studies appraised with MMAT, most had clearly stated research questions and appropriate measurements, but nonresponse bias and sample representativeness were common concerns. Study-level domain judgments are provided as Supplementary Tables S1 and S2a,b to support transparency and reproducibility.

**Table 4:** RoB 2 domain-level summary for randomized/quasi-experimental evaluations (*n* = 10).

Domain	Low Risk	Some Concerns	High Risk
Randomization process	9 (90%)	1 (10%)	0 (0%)
Deviations from interventions	3 (30%)	7 (70%)	0 (0%)
Missing outcome data	7 (70%)	3 (30%)	0 (0%)
Measurement of outcome	10 (100%)	0 (0%)	0 (0%)
Selection of reported results	9 (90%)	1 (10%)	0 (0%)
Overall risk of bias	5 (50%)	5 (50%)	0 (0%)

**Table 5:** QUADAS-2 domain-level summary for diagnostic accuracy studies (*n* = 11).

Domain	Low Risk	Unclear	High Risk
Patient selection	6 (55%)	3 (27%)	2 (18%)
Index test	9 (82%)	2 (18%)	0 (0%)
Reference standard	10 (91%)	1 (9%)	0 (0%)
Flow and timing	7 (64%)	1 (9%)	3 (27%)

**Table 6:** MMAT domain-level summary for implementation and service delivery studies (*n* = 32).

Domain	Yes	Can’t tell	No
Clear research question	29 (91%)	3 (9%)	0 (0%)
Relevant sampling strategy	21 (66%)	8 (25%)	3 (9%)
Representative sample	18 (56%)	10 (31%)	4 (13%)
Appropriate measurements	23 (72%)	7 (22%)	2 (6%)
Low nonresponse bias	15 (47%)	12 (38%)	5 (16%)

## 4. Discussion

### 4.1. Summary of Principal Findings

This systematic review identified 53 primary studies evaluating POC/near-patient pediatric HIV diagnostics and digital monitoring innovations. The evidence base was dominated by sub-Saharan Africa (39/53), with a smaller but informative Asia–Pacific subset (12/53). Across study designs, the most consistently reported benefits of POC EID and near-patient VL were reductions in turnaround time and earlier clinical action, especially when implementation included clear workflows, quality assurance, and connectivity or tracking systems. However, geographic, supply chain, and human resource constraints—particularly in dispersed Asia–Pacific settings—remain critical determinants of real-world impact and require explicit program design and evaluation [13,14,23,36].

### 4.2. Why the Evidence Base Is Africa-Heavy and What It Means for Asia–Pacific Programs

The geographic skew towards African studies reflects the global distribution of pediatric HIV burden and the concentration of donor-supported implementation science in high-burden settings [4,20]. For Asia–Pacific decision-makers, this creates a transferability challenge: technologies may be generalizable (assay chemistry and analytical performance), but implementation effects depend on the local health system context—ANC coverage, laboratory networks, geography, stigma, and models of pediatric care—as demonstrated by cross-country cascade analyses showing that gaps in PMTCT program coverage vary substantially across settings and are critical determinants of pediatric HIV outcomes [58]. Our findings should therefore be interpreted through regional constraints and opportunities. For example, cohort evidence indicates that pediatric ART coverage and viral suppression remain heterogeneous across Asia–Pacific settings, reinforcing the need for context-specific cascade diagnostics and differentiated service delivery [19,57]. Similarly, qualitative barriers to PMTCT and infant testing documented in China—such as service fragmentation and stigma—suggest that improvements in testing technology alone will not close diagnostic gaps without parallel interventions addressing demand, confidentiality, and trust [15].

#### Distinguishing Asia–Pacific Evidence from Extrapolated Findings

Given the geographic concentration of the evidence base in sub-Saharan Africa, it is important to explicitly categorize the conclusions of this review according to the strength and origin of the supporting evidence. We distinguish three tiers.

Firstly, conclusions directly supported by Asia–Pacific studies. These include: the feasibility and effectiveness of POC EID (Xpert HIV-1 Qual) in Myanmar and Papua New Guinea, demonstrated in a pragmatic cluster-randomized trial [40]; the role of systemic barriers including stigma, fragmented service delivery, and delayed result feedback in weakening PMTCT and EID outcomes in China [15]; challenges of retention and family-centered care in Cambodia [44]; and smaller regional diagnostic performance evaluations within the included Asia–Pacific study set. Together, these findings provide direct, though still limited, evidence for regional program design.

Secondly, findings supported by robust multi-setting evidence with reasonable expectation of transferability. Diagnostic accuracy of POC EID platforms (e.g., GeneXpert HIV-1 Qual, Alere q) has been established across diverse settings, and assay analytical performance is expected to be consistent across regions [22,42,43]. Similarly, the principle that shorter turnaround times improve cascade completion is supported by sufficiently diverse evidence to warrant generalization, although the magnitude of benefit may vary with local health system characteristics.

Thirdly, findings for which transferability to Asia–Pacific settings remains uncertain and requires context-specific evaluation. These include: community health worker-supported models for EID uptake [49]; conditional cash transfer approaches [38]; digital adherence and psychosocial support interventions evaluated predominantly outside the Asia–Pacific region [26,27]; and cost-effectiveness estimates derived from non-regional settings [59]. For these interventions, we recommend pilot evaluation in Asia–Pacific settings before programmatic adoption, with attention to local implementation barriers, cultural acceptability, and health system financing models.

This tiered framework should guide regional decision-makers in distinguishing evidence that can be applied with reasonable confidence from evidence that requires local adaptation and evaluation.

### 4.3. Implementation Design Considerations for Scale-Up

Implementation choices determine whether innovations close gaps or create new inequities. Based on the included evidence and programmatic guidance, four design considerations are particularly salient for the Asia–Pacific region: (i) Test placement and patient flow: POC EID yields the greatest impact when placed at high-volume, high-yield touchpoints (maternity wards, immunization clinics, malnutrition services) and when workflows minimize repeat visits for results [23,55]; a multi-country observational evaluation of routine POC EID implementation across eight African countries further demonstrated that decentralizing testing to lower-level facilities substantially reduced turnaround times and improved cascade completion, reinforcing the operational impact of strategic test placement [60]. (ii) Network thinking rather than single-device thinking: For VL monitoring and EID, hybrid models are often necessary—POC at high-volume sites and DBS or plasma referral testing for low-volume facilities. This requires specimen transport, instrument utilization governance, and cross-program coordination when multi-disease platforms are used [13,14]. (iii) Quality assurance and confirmatory testing: The clinical risk of misclassification in infants, while rare in well-run programs, has severe implications. Programs therefore require external quality assurance, clear retesting protocols, and mechanisms to manage discordant results [22,24]. (iv) Human resources and task-shifting: Task-shifting can increase throughput and reduce clinician bottlenecks, but requires training, supervision, and scope-of-practice alignment. Evidence from community health worker-supported models indicates that well-designed community linkage can improve the uptake and completion of EID steps [49].

These implementation design principles are supported primarily by evidence from sub-Saharan Africa; their applicability in Asia–Pacific settings—where health workforce structures, regulatory environments, and community health systems may differ substantially—should be validated through regional implementation research.

### 4.4. Digital Monitoring as a Force Multiplier

In this review, digital interventions were not a stand-alone solution; rather, they increased the probability that diagnostic innovations translated into clinical action. Digital tools can (i) reduce the “diagnostic limbo” created by paper-based specimen tracking and fragmented result return systems [7,14,53], (ii) support adherence monitoring and psychosocial well-being among children and adolescents when paired with human support [25,26,27], and (iii) enable continuous program performance monitoring through dashboards and cohort monitoring approaches [7,14]. These features align with WHO strategic information guidance, which emphasizes that data use—not data collection alone—is essential for improving quality, including pediatric VL suppression [7].

### 4.5. Policy and Research Implications

While POC testing can increase upfront per-test and implementation costs, it may also shorten delays and reduce avoidable losses along the diagnostic pathway. Notably, no published cost-effectiveness evaluations of POC EID or near-patient VL testing specific to Asia–Pacific pediatric settings were identified in this review; regional investment cases therefore rely on extrapolation from non-regional analyses and broader LMIC economic frameworks [59], representing a critical evidence gap.

### 4.6. Strengthening Pediatric Case-Finding in Low-Prevalence and Concentrated Epidemics

Several Asia–Pacific countries have lower generalized HIV prevalence but concentrated epidemics and substantial geographic inequities. In such contexts, broad population screening may be inefficient, and programs often require targeted pediatric case-finding strategies. Evidence and guidance support systematic testing in high-yield child health entry points (inpatient wards, malnutrition programs, tuberculosis clinics, and immunization services) and use of index case testing to identify undiagnosed children in households of adults living with HIV [55,56]. Operational experience also indicates that even modest increases in pediatric testing coverage can yield meaningful gains when combined with linkage systems; for example, national reports have described substantial increases in pediatric testing and identification in Malawi after programmatic efforts to expand child-focused testing approaches [61]. For adolescents, policy-guided self-testing and caregiver-assisted approaches may complement facility testing, but they must be implemented with robust linkage pathways and protections for confidentiality and consent [18,48].

### 4.7. Limitations

This review has several limitations that should inform interpretation of the findings. Firstly, the evidence base is heterogeneous across technologies, study designs, and outcomes, limiting the feasibility of de novo meta-analysis. We therefore relied on narrative synthesis and drew on existing pooled evidence when available [36]; this approach carries the risk of selective emphasis, which we mitigated through structured synthesis organized around the cascade framework (Figure 2) and transparent risk-of-bias reporting. Secondly, the risk-of-bias appraisals identified important methodological limitations across the included studies. Among QUADAS-2-appraised diagnostic accuracy studies (*n* = 11), 45% had unclear or high risk of bias for patient selection and 36% for flow and timing. Among MMAT-appraised implementation studies (*n* = 32), only 56% demonstrated representative sampling and only 47% were judged to have low nonresponse bias, raising the possibility that reported operational outcomes overestimate effectiveness under routine conditions. The predominantly “some concerns” ratings in RoB 2 appraisals (*n* = 10) reflect the inherent challenges of blinding in pragmatic implementation trials but nonetheless warrant cautious interpretation of effect sizes. Thirdly, the Asia–Pacific evidence base remains comparatively small (12 of 53 primary studies), and several included studies from the region were conducted in controlled or high-support environments, potentially overestimating effectiveness under routine scale-up conditions. Fourthly, program evaluations frequently reported operational outcomes without standardized definitions (e.g., different thresholds for “timely” ART initiation—within 7, 14, 30, or 60 days), complicating cross-study comparisons. Fifthly, the search window extension from 2015 to 2012 was a post-registration protocol deviation; although made before search execution, it was not prospectively updated in the INPLASY registry. Sixthly, the near-complete absence of published cost-effectiveness data for POC pediatric HIV diagnostics in Asia–Pacific settings represents a critical evidence gap, as regional investment cases currently rely on extrapolation from African cost analyses that may not reflect local cost structures or health system financing models.

### 4.8. Practical Checklist for Implementers

Based on the evidence synthesis above, we propose a practical implementation checklist for program managers planning POC EID and/or near-patient VL implementation in Asia–Pacific settings (Table 7). The evidence basis labels are intended to show how each item is justified, not to imply a formal GRADE-style recommendation: Strong evidence indicates support from multiple studies including at least one randomized or quasi-experimental evaluation; moderate evidence indicates support from observational or implementation studies; programmatic guidance indicates recommendations anchored primarily in WHO/UNICEF/ PEPFAR or similar normative frameworks; and expert consensus indicates operational recommendations for which direct comparative evidence remains limited.

## 5. Conclusions

POC and near-patient HIV diagnostics—particularly virological testing for EID and VL monitoring—can shorten time-to-result and time-to-treatment action in pediatric programs, though the strength of this evidence varies by outcome and is derived predominantly from sub-Saharan African settings. Implementation success is conditional on workflow redesign, quality assurance, and data systems that ensure results lead to clinical action; technology deployment without these enabling elements has not consistently demonstrated patient-level benefit. The quality of the evidence base—with notable limitations in patient selection, flow/timing, sample representativeness, and nonresponse bias across the appraised studies—warrants cautious interpretation of reported effect sizes, particularly when extrapolating from controlled evaluations to routine scale-up conditions. Because Asia–Pacific evidence remains limited (12 of 53 included studies) and the region is operationally diverse, regional programs should prioritize generating context-specific implementation evidence—including cost-effectiveness analyses and pragmatic evaluations embedded in routine systems—alongside the adaptation of established lessons from higher-burden settings. Particular attention is needed to address feasibility, equity, stigma reduction, and sustainable financing in the diverse health system environments of the Asia–Pacific region [7,19,23,36].

## Figures and Tables

**Table 1:** Included studies (*n* = 53) by region and innovation category (from Supplementary Table S3).

Region	Digital/Systems	EID	Other	Viral Load
Africa	10	20	4	8
Americas/Caribbean	0	1	0	0
Asia–Pacific	1	7	3	1
Multi-country/Global	−1	1	−1	−1

**Table 2:** Summary of diagnostic and digital innovations for pediatric HIV EID and monitoring (implementation lens).

Innovation Domain	Technology/Workflow	Primary Use Case	Programmatic Advantages	Key Limitations/Requirements	Asia–Pacific Implementation Notes
POC virological EID	Cartridge-based HIV-1 qualitative NAT (e.g., Xpert HIV-1 Qual)	Infant diagnosis at birth and/or 4–6 weeks	Same-day results; reduced turnaround; can increase timely ART initiation when linked to action [23,36,40]	Requires training, QA/EQA, confirmatory testing pathways, uninterrupted cartridges, and instrument uptime [22,24]	Prioritize maternity and high-volume child health clinics; use hybrid models for remote sites with referral testing [12,13]
POC virological EID	Dedicated POC NAT platforms (e.g., Alere q/m-PIMA class)	Decentralized EID in clinics without full molecular laboratories	High agreement with reference PCR in evaluations; simplified workflow [22,42]	Lower throughput than central labs; needs calibration and QA; connectivity may be limited [13,22]	Consider where sample referral times are long; budget for maintenance and staff turnover; plan connectivity for result return [7,14]
Birth testing	Birth POC testing integrated into maternity services	Rapid identification of in utero infection	Earlier detection than 6-week testing; supports rapid ART initiation [39]	Must be paired with repeat testing; confirmatory testing protocols required [16,37]	Use where loss to follow-up between birth and 6 weeks is common; align with EMTCT roadmap and postnatal follow-up systems [12]
DBS workflow optimization	DBS collection, transport, and lab optimization + digital tracking	EID and VL in settings using centralized laboratories	Leverages centralized capacity; supports remote geography; lower cold-chain requirements	Transport delays and batching can erase clinical benefit; result return often weakest link [37,44]	Implement end-to-end tracking (specimen → result → action); monitor turnaround time distributions, not averages [7,14]
Near-patient VL	Clinic-based VL platforms (e.g., SAMBA-II; POC VL class)	Routine and targeted VL monitoring in children/adolescents	Shortens time to action; supports adherence interventions and regimen switching [45,46]	Requires clear clinical algorithms and reliable supply chain; careful site selection [13,47]	Deploy to high-burden sites; integrate with differentiated service delivery models for stable vs. high-risk children [19]
Multi-disease platform integration	Integrating HIV assays on existing GeneXpert/TB platforms	Shared instrument network for TB and HIV assays	Potentially efficient use of existing assets; reduces need for parallel systems [14]	Requires governance for scheduling, cartridge forecasting, and maintenance; risk of competition between programs [14]	Highly relevant where GeneXpert networks exist; establish cross-program MoUs and performance dashboards [7,14]
Digital monitoring	Electronic registers, connectivity middleware, dashboards	Improve result delivery and cascade visibility	Reduces missed steps; enables performance management; supports QA and supervision [7,14]	Requires infrastructure, governance, data protection, and training; risk of “data without action” [7]	Start with a small set of actionable indicators (EID result return, ART initiation time, VL action time); scale iteratively [7,19]
Adolescent support and case finding	mHealth adherence/psychosocial support; self-testing; index case testing	Retention, adherence, and diagnosis in older children/adolescents	Can improve engagement when combined with human support; expands case-finding [18,26,27,48]	Confidentiality and consent issues; linkage pathways must be defined [18,48]	Align with national policies and child protection; ensure youth-friendly linkage and differentiated services [19]

**Table 3:** Cascade gaps and program responses relevant to Asia–Pacific pediatric HIV diagnostics and monitoring.

Cascade Step	Typical Programmatic Gap	Digital/QA Enablers	Suggested Monitoring Indicators
Maternal testing	Missed ANC visits; late diagnosis during breastfeeding	Electronic ANC registers; reminder systems; linkage tracking [7]	% pregnant women tested; % retested where indicated; partner testing uptake
EID testing	Delayed sample transport; long turnaround; missed testing windows	Specimen/result tracking dashboard; EQA participation [7,22]	Median days sample-to-result; % results returned ≤28 days; % HIV-exposed infants tested at birth/6 weeks
Linkage to ART	Loss to follow-up after diagnosis; delayed ART start	Line lists for positives; CHW linkage; appointment tracking [7,49]	% ART initiation ≤7/30/60 days; % retained at 6/12 months
VL monitoring	Low VL coverage; delayed action on failure	Automated VL alerts; cohort monitoring; adherence support tools [7,26]	% on ART with VL test in last 12 months; time VL→action; regimen switch rate after failure
Sustained suppression	Adherence barriers; psychosocial issues in adolescents	Adherence dashboards; youth-friendly services monitoring [19]	% suppressed (<1000 copies/mL); % resuppressed after adherence intervention; appointment adherence

Note: The “Innovation(s) that address the gap” column has been removed from Table 3 to reduce redundancy with Table 2. See Table 2 for corresponding innovation-level details and implementation notes.

**Table 7:** Practical checklist for POC EID and near-patient VL implementation in Asia–Pacific settings.

No.	Checklist Item and Key Considerations	Evidence Basis (Supporting References)
1	Define the target patient pathway and clinical decision point before instrument placement: who is tested, where the result should return, and what same-day or next-step action is expected.	Programmatic guidance [7,16,17]
2	Place POC instruments at high-volume, high-yield touchpoints (e.g., maternity wards and child health services) and define a referral pathway for low-volume sites.	Strong evidence [23,39,60]
3	Build a results-to-action protocol, including confirmatory testing rules, repeat-testing schedules, and clear VL thresholds linked to treatment action.	Programmatic guidance [7,16,17]
4	Establish a quality assurance system: EQA participation, competency assessment, supervision, and written protocols for discordant or unexpected results.	Moderate evidence [21,22,23,24]
5	Ensure supply chain resilience through cartridge forecasting, buffer stocks, maintenance contracts, and contingency plans for downtime or stock-outs.	Moderate evidence [13,14,21,22]
6	Implement connectivity and tracking where feasible: electronic test ordering, automated result delivery, and dashboards or line lists for overdue results.	Moderate evidence [7,14,53]
7	Integrate targeted pediatric case-finding strategies, including child health platform testing, index case testing, and age-appropriate self-testing policies with explicit linkage pathways.	Moderate evidence [17,18,48,55,56]
8	Plan human resources and supportive supervision: training curricula, competency checks, refresher mentoring, and clear scope of practice for nurses, laboratorians, and community workers.	Moderate evidence [19,22,49]
9	Monitor equity and coverage during scale-up by geography, age band, and service delivery point, not only by aggregate testing volume.	Programmatic guidance [7,12,19]
10	Budget for sustainability and evaluate during scale-up, including total cost of ownership, throughput, maintenance, and implementation outcomes.	Programmatic guidance [13,21,59]

The checklist is intended as a program design aid for adaptation and local evaluation, especially when evidence is being transferred from sub-Saharan African settings to operationally diverse Asia–Pacific contexts.

## Data Availability

No new data were created or analyzed in this study.

## Supplementary Materials

The following supporting information can be downloaded at: https://www.mdpi.com/article/10.3390/diagnostics16091306/s1, Prisma 2020 Checklist; Supplementary Text S1: Full search strategies for all databases; Supplementary Tables S1 and S2a,b: Study-level RoB 2, QUADAS-2, and MMAT matrices; Supplementary Table S3: Characteristics of included primary studies (*n* = 53); Supplementary Table S4: Contextual sources retained for narrative synthesis (*n* = 3); Supplementary Table S5: PRISMA 2020 checklist (included in Supplementary Text S1).
