# Supplementary material for: Closing Diagnostic Gaps in Pediatric HIV: Innovations in Point-of-Care and Digital Monitoring with an Asia–Pacific Implementation Lens—A Systematic Review
Source: Diagnostics (Basel). 2026 Apr 27;16(9):1306. doi: 10.3390/diagnostics16091306 (PMC13164136; doi:10.3390/diagnostics16091306)
Supplement: Supplementary file 1 [file diagnostics-16-01306-s001.zip › Supplementary_Tables.pdf]

Supplementary Material

Table S1. Study-level risk-of-bias matrix for randomized and quasi-experimental evaluations (RoB 2; n = 10).

Study	Randomization process	Deviations from intended interventions	Missing outcome data	Measurement of outcome	Selection of reported results	Overall
Mohamed et al., 2023	Low	Some Concerns	Some Concerns	Low	Low	Some Concerns
Jani et al., 2018	Some Concerns	Some Concerns	Low	Low	Some Concerns	Some Concerns
Technau et al., 2017	Low	Some Concerns	Low	Low	Low	Low
Mwenda et al., 2018	Low	Low	Low	Low	Low	Low
Phillips et al., 2020	Low	Some Concerns	Some Concerns	Low	Low	Some Concerns
Ibrahim et al., 2017	Low	Low	Low	Low	Low	Low
Sawadogo et al., 2022	Low	Low	Low	Low	Low	Low
Mapangisana et al., 2021	Low	Some Concerns	Low	Low	Low	Low
Jani et al., 2025	Low	Some Concerns	Some Concerns	Low	Low	Some Concerns
Lwilla et al., 2025	Low	Some Concerns	Low	Low	Low	Some Concerns

Table S2a. QUADAS-2 appraisal of diagnostic accuracy studies included in the review (n = 11).

Study	Patient selection	Index test	Reference standard	Flow and timing	Applicability concerns
Murray et al., 2017	Low	Low	Low	Low	Unclear
Hsiao et al., 2016	Unclear	Low	Low	Low	Low
Migamba et al., 2024	Low	Low	Low	High	Unclear

Agarwal et al., 2020	High	Low	Low	Low	Low
Zhang et al., 2019	Low	Unclear	Low	Low	Unclear
Peter et al., 2017	Unclear	Low	Low	High	Low
Dunning et al., 2017	Low	Low	Unclear	High	Low
Dora et al., 2020	Unclear	Low	Low	Unclear	Low
Nguyen et al., 2015	Low	Unclear	Low	Low	Low
Muratova et al., 2021	High	Low	Low	Low	Low
Ayouba et al., 2018	Low	Low	Low	Low	Unclear

Table S2b. Mixed Methods Appraisal Tool (MMAT, 2018) assessment of implementation, service-delivery, and digital-support studies (n = 32).

Study	Clear research question	Relevant sampling	Representative sample	Appropriate measurements	Low nonresponse bias
Odeny et al., 2014	Yes	Can’t tell	Yes	Yes	Can’t tell
Seidenberg et al., 2012	Yes	Yes	Can’t tell	Yes	Yes
Finocchario-Kessler et al., 2014	Yes	Yes	Yes	Can’t tell	Yes
Wexler et al., 2017	Yes	Yes	Yes	Yes	Can’t tell
Boeke et al., 2021	Yes	Yes	Yes	Yes	No
Sutcliffe et al., 2017	Can’t tell	Yes	Yes	Can’t tell	Yes
Gill et al., 2017	Yes	Yes	Yes	Yes	Can’t tell
Finocchario-Kessler et al., 2018	Can’t tell	Yes	Yes	Yes	No
Fraser et al., 2018	Can’t tell	No	Yes	Can’t tell	Can’t tell
Willcox et al., 2019	Yes	Can’t tell	Yes	Can’t tell	Can’t tell
Sibanda et al., 2015	Yes	Yes	Yes	Can’t tell	Yes

Jean et al., 2020	Yes	Yes	Yes	Yes	Can’t tell
Torres et al., 2020	Yes	Yes	No	Yes	Can’t tell
Khumalo et al., 2020	Yes	Yes	Yes	Yes	No
Technau et al., 2017	Yes	Yes	Can’t tell	No	Yes
Buzdugan et al., 2016	Yes	Can’t tell	Yes	Yes	Can’t tell
Fonjungo et al., 2013	Yes	Yes	No	Yes	No
Lolekha et al., 2020	Yes	Can’t tell	Yes	Can’t tell	Yes
Lesosky et al., 2015	Yes	No	Can’t tell	Yes	Yes
Lu et al., 2021	Yes	Yes	Yes	Yes	No
Bwana et al., 2019	Yes	Yes	Can’t tell	Yes	Yes
Pilotto et al., 2018	Yes	Can’t tell	Can’t tell	Yes	Yes
Chiabi et al., 2021	Yes	No	Yes	Yes	Yes
Ananworanich et al., 2016	Yes	Can’t tell	Yes	Yes	Yes
Kiyaga et al., 2021	Yes	Can’t tell	No	Yes	Can’t tell
Sinha et al., 2019	Yes	Yes	No	Yes	Yes
Nwanja et al., 2023	Yes	Can’t tell	Can’t tell	Yes	Yes
Chetsanga et al., 2015	Yes	Yes	Yes	Yes	Can’t tell
Ali et al., 2020	Yes	Yes	Can’t tell	Yes	Can’t tell
Nordberg et al., 2021	Yes	Yes	Can’t tell	No	Yes
Hayashida et al., 2015	Yes	Yes	Can’t tell	Can’t tell	Yes
Bianchi et al., 2019	Yes	Yes	Can’t tell	Yes	Can’t tell

Table S3. Characteristics of included primary studies (n = 53).

Study (Author, Year)	Country/Setting	Population	Intervention	Comparator	Key Outcomes	Full Citation

Jani et al., 2018	Mozambique	HIV-exposed infants (6–8 weeks)	POC EID at clinics (Alere q PCR)	Central lab PCR EID	Faster result return; higher ART initiation by 12 months	Jani IV, Meggi B, Mabunda S, et al. Effect of point-of-care early infant diagnosis on antiretroviral therapy initiation and retention of patients. AIDS 2018;32(11):1453-1463. doi:10.1097/QAD.0000000000001846. PMID: 29746301.
Technau et al., 2017	South Africa	HIV-exposed neonates (birth)	POC birth HIV testing + immediate ART	Standard 6-week EID	Earlier infant HIV detection; no difference in 18-month survival	
Odeny et al., 2014	Kenya	Mothers of HIV-exposed infants	SMS reminders for EID visits/results	Standard care (no SMS)	Improved clinic return for results; earlier infant testing	Odeny TA, Bukusi EA, Cohen CR, et al. Texting improves testing: a randomized trial of two-way SMS to increase postpartum prevention of mother-to-child transmission retention and infant HIV testing. AIDS 2014;28(15):2307-2312. doi:10.1097/QAD.0000000000000409. PMID: 25313586
Seidenberg et al., 2012	Zambia	EID samples in rural clinics	SMS-based result transmission system	Paper-based result delivery	Reduced result turnaround time to clinics	Seidenberg P, Nicholson S, Schaefer M, et al. Early infant diagnosis of HIV infection in Zambia through mobile phone texting of blood test results. Bull World Health Organ 2012;90(5):348-356. doi:10.2471/BLT.11.100032. PMID: 22589568
Finocchario-Kessler et al., 2014	Kenya	HIV-exposed infants & mothers	HITSystem (digital EID tracking with SMS)	Standard EID process	Improved EID retention and faster ART initiation for infants	Finocchario-Kessler S, Gautney BJ, Khamadi S, et al. If you text them, they will come: using the HIV infant tracking system to improve early infant diagnosis quality and retention in Kenya. AIDS 2014;28(Suppl 3):S313-S321. doi:10.1097/QAD.0000000000000332. PMID:

						24991904
Wexler et al., 2017	Kenya	Early infant diagnosis program data	Implementation of HITSystem (retrospective analysis)	Pre-implementation data	Shorter median turnaround times for EID results	Wexler C, Cheng AL, Gautney B, et al. Evaluating turnaround times for early infant diagnosis samples in Kenya from 2011-2014: a retrospective analysis of HITSystem program data. PLoS One 2017;12(8):e0181005. doi:10.1371/journal.pone.0181005. PMID: 28796791
Boeke et al., 2021	Multi-country (6 African countries)	HIV-exposed infants in national programs	POC EID rollout (m-PIMA/GeneXpert devices)	Historical standard EID	Increased timely result return; quicker ART initiation	Boeke CE, Joseph J, Wang M, et al. Point-of-care testing can achieve same-day diagnosis for infants and rapid ART initiation: results from government programmes across six African countries. J Int AIDS Soc. 2021;24(3):e25677. doi:10.1002/jia2.25677. PMID: 33745218.
Mwenda et al., 2018	Malawi (7 health facilities)	HIV-exposed infants (program data)	POC EID implemented vs. centralized EID	Standard centralized EID	Dramatically reduced result return time; improved early ART initiation	Mwenda R, Fong Y, Magombo T, et al. Significant patient impact observed upon implementation of point-of-care early infant diagnosis technologies in an observational study in Malawi. Clin Infect Dis 2018;67(5):701-707. doi:10.1093/cid/ciy169. PMID: 29490026.
Murray et al., 2017	South Africa	Infants born to HIV+ mothers	POC EID (Xpert HIV-1 Qual)	Lab PCR (reference standard)	High sensitivity (~95%) & specificity (~99%); faster results	Murray TY, Sherman GG, Nakwa FL, MacLeod WB, Sipambo N, Velaphi S, et al. Field evaluation of performance of Alere and Cepheid qualitative HIV assays for pediatric point-of-care testing in an academic hospital in Soweto, South Africa. J Clin Microbiol. 2017;55(11):3227-3235. doi:10.1128/JCM.01021-17. PMID: 28855305.
Dunning et al., 2017	South Africa	Newborns at birth and	POC EID at birth (Alere q HIV-1/2	Standard 6-week PCR EID	Feasible early detection	Dunning L, Kroon M, Hsiao NY, Myer L. Field evaluation of HIV point-of-care testing for

		6 weeks	Detect)		at birth; identified infections missed by 6-week EID	early infant diagnosis in Cape Town, South Africa. PLoS One. 2017;12(12):e0189226. doi:10.1371/journal.pone.0189226. PMID: 29232371.
Bianchi et al., 2019	Mozambique	Infants and caregivers in clinics	POC viral load testing (Abbott m-PIMA)	Central lab viral load	Results available same day; enabled immediate counseling and interventions	
Sutcliffe et al., 2017	Zambia	Dried blood spot EID network	Introduction of SMS/GPRS printers for results	Traditional courier results	Significantly reduced time to result notification at clinics	Sutcliffe CG, van Dijk JH, Hamangaba F, et al. Use of mobile phones and text messaging to decrease the turnaround time for early infant HIV diagnosis and notification in rural Zambia: an observational study. BMC Pediatr 2017;17(1):66. doi:10.1186/s12887-017-0822-z. PMID: 28270134
Migamba et al., 2024	Uganda	Infants in EID program	Decentralized POC EID at hospitals (GeneXpert)	Central lab PCR	Reduced turnaround from weeks to days; more results received within 1 month	Migamba SM, Nyombi TN, Nsubuga EJ, et al. Rapid antiretroviral therapy initiation following rollout of point-of-care early infant diagnosis testing, Uganda, 2018-2021. AIDS Res Ther. 2024;21:31. doi:10.1186/s12981-024-00613-8. PMID: 38750529.
Gill et al., 2017	Lesotho	HIV-exposed infants at maternity sites	Immediate birth PCR testing (at delivery, lab-based)	Standard 6-week testing	Higher early HIV detection at birth; earlier treatment initiation	Gill MM, Hoffman HJ, Mokone M, Tukei VJ, Nchephe M, Phalatse M, et al. Assessing very early infant diagnosis turnaround times: findings from a birth testing pilot in Lesotho. AIDS Res Treat. 2017;2017:2572594. doi:10.1155/2017/2572594. PMID: 29410914.

Agarwal et al., 2020	India	Infants born to HIV+ mothers	POC EID at point-of-care (GeneXpert)	Referral lab PCR testing	Improved access to testing in remote areas; faster result delivery	
Zhang et al., 2019	China	Infants in rural areas	POC PCR diagnostic platform (decentralized testing)	Central lab testing	Enabled testing closer to patients with reduced wait times; maintained high accuracy	
Finocchario-Kessler et al., 2018	Kenya	Mother-infant pairs in EID care	Enhanced HITSystem + bidirectional SMS	Standard care (no digital system)	Increased completion of EID steps; more infants started on ART by 3 months	Finocchario-Kessler S, Gautney B, Cheng A, et al. Evaluation of the HIV Infant Tracking System (HITSystem) to optimise quality and efficiency of early infant diagnosis: a cluster-randomised trial in Kenya. Lancet HIV 2018;5(12):e696-e705. doi:10.1016/S2352-3018(18)30245-5. PMID: 30309787
Peter et al., 2017	Kenya	HIV+ children needing VL check	POC viral load test (SAMBA II)	Standard lab viral load	High concordance with lab results; enabled same-day clinical action	
Hsiao et al., 2016	South Africa	Infant dried blood samples (laboratory study)	Alere q HIV-1/2 Detect (POC PCR)	Roche COBAS PCR (lab standard)	Sensitivity ~98–100% on DBS; reliable EID performance at point-of-care	Hsiao NY, Dunning L, Kroon M, Myer L. Laboratory evaluation of the Alere q point-of-care system for early infant HIV diagnosis. PLoS One. 2016;11(3):e0152672. doi:10.1371/journal.pone.0152672. PMID: 27032094.

Fraser et al., 2018	Cameroon	HIV-exposed infants in clinics	SMS printer system for EID results delivery	Paper-based results	Marked improvement in result delivery time; higher caregiver notification rates	Vojnov L, Markby J, Boeke C, et al. Impact of SMS/GPRS printers in reducing time to early infant diagnosis compared with routine result reporting: a systematic review and meta-analysis. J Acquir Immune Defic Syndr. 2017;76(5):522-526. doi:10.1097/QAI.0000000000001526. PMID: 28825941.
Willcox et al., 2019	Malawi	Primary care clinics (EID services)	Electronic result system (LIS/SMS)	Prior manual results system	Decreased turnaround times; improved linkage of HIV+ infants to care	
Sibanda et al., 2015	Zimbabwe	National EID program	Motorbike courier + SMS notification for DBS results	Standard postal/courier system	Faster DBS transport & result communication; reduced diagnostic delays	
Jean et al., 2020	Haiti	Infants in PMTCT program	POC EID pilot (GeneXpert) in clinics	Central lab PCR	~95% results returned same-day; caregivers informed much sooner than before	
Torres et al., 2020	Mozambique	Children (1–14 years) on ART	POC viral load monitoring (Abbott m-PIMA)	Lab viral load (≈6-week wait)	~80% received same-day results; enabled earlier regimen changes for	

					failures	
Khumalo et al., 2020	Eswatini	HIV+ infants & children in care	Digital patient tracking + POC diagnostics	Standard care before	Improved retention in care; timelier confirmatory testing & treatment initiation	Khumalo PN, Sacks E, Chouraya C, Tsabedze B, Masuku T, Nyoni G, et al. The cascade of care from routine point-of-care HIV testing at birth: results from an 18-months pilot program in Eswatini. J Acquir Immune Defic Syndr. 2020;84(Suppl 1):S22-S27. doi:10.1097/QAI.0000000000002380. PMID: 32520911.
Technau et al., 2017	South Africa	Newborns of HIV+ mothers	Universal birth PCR testing (lab-based)	Postnatal 6-week testing	Birth PCR testing feasible at urban hospital level; identified successes and operational challenges in early infant identification and linkage to care	Technau KG, Kuhn L, Coovadia A, et al. Improving early identification of HIV-infected neonates with birth PCR testing in a large urban hospital in Johannesburg, South Africa: successes and challenges. J Int AIDS Soc. 2017;20(1):21436. doi:10.7448/IAS.20.01/21436. PMID: 28406596.
Buzdugan et al., 2016	Zimbabwe	HIV-exposed infants (follow-up phase)	SMS reminders for EID result pickup	No SMS (standard care)	Higher proportion of caregivers returned for EID results	
Fonjungo et al., 2013	Ethiopia	Infants in remote areas	Decentralized EID (local PCR labs added)	Centralized lab EID only	Increased EID coverage in rural sites; shorter result return time	Fonjungo PN, Girma M, Melaku Z, et al. Field expansion of DNA polymerase chain reaction for early infant diagnosis of HIV-1: the Ethiopian experience. Afr J Lab Med. 2013;2(1):31. doi:10.4102/ajlm.v2i1.31. PMID: 26855901.

Mapangisana et al., 2021	Zimbabwe	Children on ART (5–15 years)	POC viral load at clinics (Abbott m-PIMA)	Central lab viral load	~98% got same-day results vs none with lab; time to action cut by about 2 months	Mapangisana T, Machekano R, Kouamou V, et al. Viral load care of HIV-1 infected children and adolescents: a longitudinal study in rural Zimbabwe. PLoS One. 2021;16(1):e0245085. doi:10.1371/journal.pone.0245085. PMID: 33444325.
Lolekha et al., 2020	Thailand	HIV-exposed infants (provincial level)	Online result dispatch system for EID	Traditional paper dispatch	Result notification time halved (~4 weeks to 2 weeks); improved caregiver result receipt	Lolekha R, Pavaputanon P, Puthanakit T, et al. Implementation of an active case management network to identify HIV-positive infants and accelerate the initiation of antiretroviral therapy, Thailand 2015 to 2018. J Int AIDS Soc. 2020;23(2):e25450. doi:10.1002/jia2.25450. PMID: 32107884.
Lesosky et al., 2015	South Africa	HIV+ children on ART	Frequent POC VL monitoring (e.g., monthly tests)	Standard quarterly VL testing	Earlier detection of virologic failure; more timely interventions	
Phillips et al., 2020	Lesotho	HIV-exposed infants (community outreach)	Combined POC EID + peer tracking	Standard facility-based EID	Near-complete testing coverage; most HIV+ infants on ART within 1 week	
Lu et al., 2021	China	Mother-infant pairs in PMTCT program	Digital case management system (tracking)	No digital tracking (historical)	Improved follow-up testing rates; fewer infants	

					lost to follow-up	
Dora et al., 2020	India	Infants at delivery (birth)	Rapid POC DNA test (Truenat) at birth	Standard PCR after birth	All HIV+ infants started ART <1 week old; greatly shortened result waiting time	
Bwana et al., 2019	Kenya	HIV+ adolescents on ART	POC viral load at youth clinics	Central lab viral load	More results received and counseling done; no significant 6-month VL difference	Bwana P, Ageng'o J, Danda J, et al. Performance and usability of mPIMA HIV 1/2 viral load test in point of care settings in Kenya. J Clin Virol. 2019;121:104202. doi:10.1016/j.jcv.2019.104202. PMID: 31715524.
Pilotto et al., 2018	Papua New Guinea	Children & adolescents on ART	POC viral load testing (GeneXpert platform)	Samples sent to external lab	Treatment failures identified much faster locally; improved management in remote areas	
Sawadogo et al., 2022	Burkina Faso	Infants at immunization clinics	POC EID offered alongside routine vaccines	Lab-referral EID (previous approach)	EID uptake ~90% at 6–8 weeks (vs ~50% before); reduced loss to follow-up	
Chiabi et al., 2021	Cameroon	EID clinics and laboratories	SMS alerts to health workers for positive EID results	Standard result communication	Clinics notified within 1 day vs ~4 weeks;	

					faster family contact & treatment initiation	
Ananworanich et al., 2016	Thailand	HIV-infected infants (research cohort)	Very early infant diagnosis & immediate ART	Standard 6-week EID	Infections detected ~4 weeks earlier; ART started at <1 month of age	
Kiyaga et al., 2021	Uganda	EID facilities (national program)	EID process tracking with performance dashboards	Pre-implementation baseline	Improved result return rates; fewer infants lost before treatment initiation	Kiyaga C, Narayan V, McConnell I, Elyanu P, Kisaakye LN, Joseph E, et al. Uganda's 'EID Systems Strengthening' model produces significant gains in testing, linkage, and retention of HIV-exposed and infected infants: an impact evaluation. PLoS One. 2021;16(2):e0246546. doi:10.1371/journal.pone.0246546. PMID: 33539425.
Sinha et al., 2019	India	Infants in northern India	More decentralized EID labs (regional testing)	Prior centralized-only system	Increased EID testing coverage; reduced median age at diagnosis	
Nwanja et al., 2023	Nigeria	Community outreach for EID	POC EID at community sites	Facility-based EID only	Reached infants missing facility testing; increased HIV+ infant case finding	Nwanja E, Nwaokoro P, Akpan U, et al. Improved access to HIV diagnosis and linkage to antiretroviral therapy among children in Southern Nigeria: a before-after study. BMC Pediatr. 2023;23(1):253. doi:10.1186/s12887-023-04050-w. PMID: 37210497.
Ibrahim et al., 2017	Botswana	Newborns at HIV testing in	Cepheid Xpert HIV-1 Qual POC test	Reference standard PCR	Cepheid Xpert HIV-1 Qual	Ibrahim M, Moyo S, Mohammed T, et al. Brief report: high sensitivity and specificity of the

		Botswana	(diagnostic evaluation)		demonstrated high sensitivity and specificity for HIV diagnosis in newborns	Cepheid Xpert HIV-1 qualitative point-of-care test among newborns in Botswana. J Acquir Immune Defic Syndr. 2017;75(5):e128-e131. doi:10.1097/QAI.0000000000001384. PMID: 28350554.
Chetsanga et al., 2015	Zimbabwe	EID program (nationwide review)	Results hotline for clinics (digital inquiry)	No hotline (historical)	Average time for clinics to receive results significantly reduced	
Nguyen et al., 2015	Vietnam	Provincial labs and clinics	Provincial EID PCR (decentralized testing)	Central lab only (before)	Wait times reduced from ~4 weeks to ~1 week; more infants tested by 2 months of age	
Muratova et al., 2021	Kazakhstan	Infants born to HIV+ mothers	Faster PCR platform implemented in-country	Older, slower PCR process	Turnaround improved; nearly eliminated diagnoses after 2 months of age	
Ali et al., 2020	Nigeria	Mother-infant pairs at EID clinics	HITSystem implementation (nationwide)	Pre-implementation baseline	Improved retention through EID steps; reduced time to infant treatment initiation	
Nordberg et al., 2021	Kenya	EID program (multiple	mHealth app for EID	Baseline paper-based	Better coordination of EID	

		facilities)	tracking (real-time data)	tracking	services; fewer infants missed for testing	
Ayouba et al., 2018	Cameroon	Infant samples (diagnostic study)	HIV PCR on dried blood spots via POC device	Whole blood testing on POC	Similar sensitivity on DBS vs whole blood; facilitated easier infant sample collection	
Hayashida et al., 2015	Cambodia	Infants in PMTCT program	Decentralized EID testing at regional labs	Previous central lab only	Increased proportion of infants tested by 3 months; shorter time to results	
Mohamed et al., 2023	Myanmar & Papua New Guinea	HIV-exposed infants (EID program)	POC EID (Xpert HIV-1 Qual) implementation	Standard centralized EID	Reduced time-to-result; increased timely result return and linkage to ART	Mohamed Y, Htay H, Gare J, et al. The effect of the Xpert HIV-1 Qual test on early infant diagnosis of HIV in Myanmar and Papua New Guinea: a pragmatic, cluster-randomised trial. Lancet HIV 2023;10(4):e220-e229. doi:10.1016/S2352-3018(23)00001-2. PMID: 36871568.
Jani et al., 2025	Mozambique & Tanzania (28 primary healthcare facilities; LIFE study)	HIV-exposed neonates (N = 6,602 enrolled; 125 HIV+ by week 12)	POC EID at birth and 4–8 weeks with immediate ART	Standard care: POC EID and ART at 4–8 weeks only	No significant 18-month composite reduction (aIRR 0.857, 95% CI 0.505–1.492); 68% relative reduction	Jani IV, Sabi I, Elsbernd K, et al. Impact of point-of-care birth test-and-treat on clinical outcomes among infants with HIV: a cluster-randomized trial in Mozambique and Tanzania. Clin Infect Dis 2025;80(5):1114-1124. doi:10.1093/cid/ciae530

					in 6-month mortality; median ART initiation 6 vs 33 days; VL suppression at 18 months 65.7% vs 29.6% (p = 0.005); poor overall suppression	
Lwilla et al., 2025	Mozambique & Tanzania (same 28 LIFE study facilities)	Women living with HIV delivering at facilities and their neonates	POC maternal VL at delivery + clinical criteria for VHT risk assessment	Clinical criteria alone; Mozambique universal ePNP, Tanzania risk-stratified ePNP	POC maternal VL substantially improved high-risk infant identification; in Tanzania improved ePNP allocation for high-risk infants; in Mozambique limited prophylaxis impact (universal ePNP) but improved risk documentation	Lwilla AF, Elsbernd K, Boniface S, et al. Impact of point-of-care maternal viral load testing at delivery on vertical HIV transmission risk assessment and neonatal prophylaxis: a cluster randomized trial. J Int AIDS Soc 2025;28(8):e70021. doi:10.1002/jia2.70021

Table S4. Contextual sources retained for narrative synthesis (n = 3).

Source	Type	Role in synthesis
Luo et al., 2022	Meta-analysis	Used to contextualize pooled evidence on point-of-care EID outcomes and ART initiation effects.
World Health Organization, 2021	Guideline	Used to contextualize infant diagnosis, ART initiation, and monitoring recommendations.
UNICEF/WHO, 2023	Framework / strategic guidance	Used to contextualize programmatic integration of EID into child health services.
