# Supplementary material for: Closing Diagnostic Gaps in Pediatric HIV: Innovations in Point-of-Care and Digital Monitoring with an Asia–Pacific Implementation Lens—A Systematic Review
Source: Diagnostics (Basel). 2026 Apr 27;16(9):1306. doi: 10.3390/diagnostics16091306 (PMC13164136; doi:10.3390/diagnostics16091306)
Supplement: Supplementary file 1 [file diagnostics-16-01306-s001.zip › Supplementary_TextS1_PRISMA.pdf]

Supplementary Material

Supplementary Text S1. Full database search strategies

PubMed/MEDLINE (run 1 November 2025; date limits 1 Jan 2012–1 Nov 2025):

((HIV[Title/Abstract] OR "human immunodeficiency virus"[Title/Abstract]) AND (infant*[Title/Abstract] OR child*[Title/Abstract] OR pediatric*[Title/Abstract] OR paediatric*[Title/Abstract] OR adolescent*[Title/Abstract]) AND ("early infant diagnosis"[Title/Abstract] OR EID[Title/Abstract] OR "HIV DNA PCR"[Title/Abstract] OR "viral load"[Title/Abstract] OR "point-of-care"[Title/Abstract] OR "near-patient"[Title/Abstract] OR POC[Title/Abstract] OR GeneXpert[Title/Abstract] OR Xpert[Title/Abstract] OR "HIV-1 Qual"[Title/Abstract] OR "Alere q"[Title/Abstract] OR m-PIMA[Title/Abstract] OR SAMBA[Title/Abstract] OR "dried blood spot"[Title/Abstract] OR DBS[Title/Abstract] OR connectivity[Title/Abstract] OR SMS[Title/Abstract] OR mHealth[Title/Abstract] OR "digital health"[Title/Abstract]))

EMBASE (via Ovid; run 1 November 2025; date limits 2012–2025):

(HIV.ti,ab OR "human immunodeficiency virus".ti,ab) AND (infant*.ti,ab OR child*.ti,ab OR pediatric*.ti,ab OR paediatric*.ti,ab OR adolescent*.ti,ab) AND ("early infant diagnosis".ti,ab OR EID.ti,ab OR "HIV DNA PCR".ti,ab OR "viral load".ti,ab OR "point-of-care".ti,ab OR "near-patient".ti,ab OR POC.ti,ab OR GeneXpert.ti,ab OR Xpert.ti,ab OR "HIV-1 Qual".ti,ab OR "Alere q".ti,ab OR m-PIMA.ti,ab OR SAMBA.ti,ab OR "dried blood spot".ti,ab OR DBS.ti,ab OR connectivity.ti,ab OR SMS.ti,ab OR mHealth.ti,ab OR "digital health".ti,ab)

Cochrane Library (run 1 November 2025; date limits 2012–2025):

(HIV OR "human immunodeficiency virus"):ti,ab,kw AND (infant* OR child* OR pediatric* OR paediatric* OR adolescent*):ti,ab,kw AND ("early infant diagnosis" OR EID OR "viral load" OR "point-of-care" OR "near-patient" OR POC OR GeneXpert OR Xpert OR "Alere q" OR SAMBA OR "dried blood spot" OR DBS OR connectivity OR SMS OR mHealth OR "digital health"):ti,ab,kw

WHO Global Index Medicus (run 1 November 2025; date limits 2012–2025):

(HIV OR "human immunodeficiency virus") AND (infant OR child OR pediatric OR paediatric OR adolescent) AND ("early infant diagnosis" OR "viral load" OR "point-of-care" OR GeneXpert OR Xpert OR "Alere q" OR SAMBA OR "dried blood spot" OR DBS OR connectivity OR SMS OR mHealth OR "digital health")

All database searches were complemented by hand-searching reference lists of included studies and targeted searches of UNAIDS, WHO, UNICEF, and PEPFAR pediatric reporting portals.

Supplementary Table S5. PRISMA 2020 checklist (completed)

Checklist items are mapped to the section(s) of the manuscript where they are addressed.

Section	Item	Checklist item (paraphrased)	Where addressed
Title	1	Identify the report as a systematic review	Title page; Abstract
Abstract	2	Structured summary of objectives, methods, results, conclusions	Abstract
Rationale	3	Describe rationale in context of existing knowledge	Introduction

Objectives	4	State review question/objectives	Introduction (Review question; Objectives)
Eligibility	5	Specify inclusion/exclusion criteria	Methods 2.2
Information sources	6	List databases and other sources searched; dates	Methods 2.3; Text S1
Search strategy	7	Provide full electronic search strategy	Supplementary Text S1
Selection process	8	Describe screening and selection process	Methods 2.4
Data collection	9	Describe data extraction process and items	Methods 2.5
Risk of bias	10	Describe tools and process for quality appraisal (RoB 2, QUADAS-2, MMAT)	Methods 2.6; Supplementary S1–S2b
Outcome measures	11	List and define all outcomes for which data were sought	Methods 2.5
Effect measures	12	Specify effect measures or synthesis approach	Methods 2.7
Synthesis methods	13	Describe synthesis approach and handling of heterogeneity	Methods 2.7
Reporting bias assessment	14	Describe methods for assessing risk of bias due to missing results	Not formally assessed; acknowledged in Discussion 4.7
Certainty assessment	15	Describe methods for assessing certainty of evidence	Not formally assessed; narrative synthesis (Methods 2.7)
Study selection results	16a	Describe results of search/selection	Results 3.1; Figure 1
Protocol deviations	16b	Describe any deviations from the protocol	No major deviations from protocol
Study characteristics	17	Cite and present included-study characteristics	Results 3.2; Supplementary Table S3
Risk of bias results	18	Present study quality appraisal results	Results 3.7; Tables 4–6; Supplementary S1–S2b
Results of individual studies	19	Summarize results of included studies	Results 3.3–3.6; Table 2–3
Results of syntheses	20a	For each synthesis, briefly summarize characteristics and results	Results 3.3–3.6; Tables 2–3
Reporting biases	21	Present assessments of risk of bias due to missing results	Not formally assessed; see Discussion 4.7
Certainty of evidence	22	Present assessments of certainty of evidence for each outcome	Not formally graded; see Discussion 4.1
Discussion	23a	Summarize main findings and strength of evidence	Discussion 4.1–4.8
Limitations	23b	Discuss limitations of evidence and review methods	Discussion 4.7
Conclusions	23c	Interpretation and	Discussion 4.8;

		implications	Conclusions
Registration and protocol	24a	Provide registration information or state not registered	Methods 2.1; registered in INPLASY (INPLASY2025110058)
Funding	25	Report funding and role	Funding statement
Competing interests	26	Declare competing interests of review authors	Conflicts of Interest statement
Data availability	27	Report which of the following are publicly available: data forms, extracted data, analytic code, other materials	Data Availability Statement
